# Amyotrophic lateral sclerosis mutant TDP-43 may cause synaptic dysfunction through altered dendritic spine function

**DOI:** 10.1242/dmm.038109

**Published:** 2019-05-17

**Authors:** Tongcui Jiang, Emily Handley, Mariana Brizuela, Edgar Dawkins, Katherine E. A. Lewis, Rosemary M. Clark, Tracey C. Dickson, Catherine A. Blizzard

**Affiliations:** Menzies Institute for Medical Research, University of Tasmania, Medical Sciences Precinct, 17 Liverpool Street, Hobart, TAS 7000, Australia

**Keywords:** TDP-43, Synapse, Dendrite spine, AMPA, Excitability

## Abstract

Altered cortical excitability and synapse dysfunction are early pathogenic events in amyotrophic lateral sclerosis (ALS) patients and animal models. Recent studies propose an important role for TAR DNA-binding protein 43 (TDP-43), the mislocalization and aggregation of which are key pathological features of ALS. However, the relationship between ALS-linked TDP-43 mutations, excitability and synaptic function is not fully understood. Here, we investigate the role of ALS-linked mutant TDP-43 in synapse formation by examining the morphological, immunocytochemical and excitability profile of transgenic mouse primary cortical pyramidal neurons that over-express human TDP-43^A315T^. In TDP-43^A315T^ cortical neurons, dendritic spine density was significantly reduced compared to wild-type controls. TDP-43^A315T^ over-expression increased the total levels of the α-amino-3-hydroxy-5-methyl-4-isoxazolepropinionic acid (AMPA) glutamate receptor subunit GluR1, yet the localization of GluR1 to the dendritic spine was reduced. These postsynaptic changes were coupled with a decrease in the amount of the presynaptic marker synaptophysin that colocalized with dendritic spines. Interestingly, action potential generation was reduced in TDP-43^A315T^ pyramidal neurons. This work reveals a crucial effect of the over-expression mutation TDP-43^A315T^ on the formation of synaptic structures and the recruitment of GluR1 to the synaptic membrane. This pathogenic effect may be mediated by cytoplasmic mislocalization of TDP-43^A315T^. Loss of synaptic GluR1, and reduced excitability within pyramidal neurons, implicates hypoexcitability and attenuated synaptic function in the pathogenic decline of neuronal function in TDP-43-associated ALS. Further studies into the mechanisms underlying AMPA receptor-mediated excitability changes within the ALS cortical circuitry may yield novel therapeutic targets for treatment of this devastating disease.

## Abstract (precis)

**Summary:** Loss of synaptic GluR1, and reduced excitability within pyramidal neurons, implicates hypoexcitability and attenuated synaptic function in the pathogenic decline of neuronal function in TDP-43-associated ALS.

## INTRODUCTION

Amyotrophic lateral sclerosis (ALS) is the most common form of adult-onset motor neuron disease. In ALS, the degeneration of cortical and spinal motor neurons results in loss of voluntary movement, progressive muscle paralysis and ultimately death within 2-5 years of diagnosis (Talbot, 2014; Zufiria et al., 2016). This disease is predominately sporadic, with only 10% of cases due to familial inheritance of an autosomal dominant mutation in genes such as *SOD1*, *C9orf72*, *FUS* or *TARDBP* (Kiernan et al., 2011; Renton et al., 2014). Similarities in pathological hallmarks and the clinical progression of both sporadic and familial forms of ALS have led to the suggestion of a commonality in the final neurodegenerative pathway. In recent years, this theory has extended to observations of altered excitability. Clinical electrophysiological studies have identified the phenomenon of cortical hyperexcitability in sporadic and familial forms of ALS, preceding both the onset of clinical symptoms and measurable lower motor neuron dysfunction in patients (reviewed by Geevasinga et al., 2016). This suggests that imbalances in motor cortex excitation are one of the earliest pathological events in the disease (Fogarty, 2018). Furthermore, cortical hyperexcitability may propagate through the corticomotor system (Menon et al., 2015; Eisen et al., 1993; Vucic et al., 2013), leading to degeneration of lower motor neurons. Yet clinical, animal model and human induced pluripotent stem cell (iPSC) studies now indicate that excitability alterations in ALS are a complex and evolving sequence of events, potentially involving both hyperexcitability and hypoexcitability of various neuron and interneuron populations that make up the cortical circuitry and varying at different disease stages (Clark et al., 2015; Geevasinga et al., 2016; Leroy and Zytnicki, 2015; White et al., 2018).

In 2006, transactive response DNA-binding protein 43 (TDP-43) was recognized as the primary protein component of intracellular ubiquitinated inclusions in the majority of ALS cases and a subset of frontotemporal lobar degeneration (FTLD) cases (Neumann et al., 2006; Geser et al., 2010). Mutations in the *TARDBP* gene, which encodes TDP-43, such as that resulting in an alanine to threonine amino acid substitution (TDP-43^A315T^), were identified in familial forms of ALS (Neumann et al., 2006). Mislocalized or mutant TDP-43 is suspected to play a major role in ALS pathogenesis (Buratti and Baralle, 2008; Sreedharan et al., 2008; Yokoseki et al., 2008), yet it is unclear how TDP-43 dysfunction or mutation is linked to altered cortical excitability. TDP-43 is thought to play a role in the synaptic connections of lower motor neurons; it is localized to the neuromuscular junction (NMJ) in mice (Narayanan et al., 2013) and is required for the development and locomotor function of the NMJ in *Drosophila* (Estes et al., 2011; Estes et al., 2013; Feiguin et al., 2009; Li et al., 2010). Mutation or expression modulation of TDP-43 also leads to impaired locomotor function and NMJ disturbances in *Caenorhabditis elegans* (Ash et al., 2010) and *Danio rerio* (Armstrong and Drapeau, 2013; Kabashi et al., 2010; Schmid et al., 2013). Interestingly, calcium channel agonists rescue this phenotype in mutant TDP-43 zebrafish, indicating that disruption to synaptic homeostasis plays a key role in the pathogenesis of mutated TDP-43 at this distal site (Armstrong and Drapeau, 2013).

However, little is known about the effect of mutant TDP-43 on excitability in upper motor neurons. Motor neurons derived from iPSCs carrying a TDP-43 mutation demonstrate a hyperexcitable phenotype that switches to hypoexcitability as the neurons mature (Devlin et al., 2015), and we previously demonstrated synaptic disturbances in the motor cortex occur early in a mouse model that expresses human TDP-43^A315T^ (Handley et al., 2017). To determine the effect of mutant TDP-43 on the development and formation of synapses in upper motor neurons *in vitro*, we derived primary cortical neuronal cultures from transgenic mouse embryos expressing human TDP-43^A315T^ under the *Prp* promoter (Wegorzewska et al., 2009) and yellow fluorescent protein (YFP) in cortical pyramidal/projection neurons under the *Thy1* promoter (Feng et al., 2000). We examined neuronal process structure, dendritic spine density, synaptic protein localization and electrophysiological excitability. This study identified that ALS-linked mutant TDP-43 (A315T mutation) has a significant pathological influence on synapse development in pyramidal cortical neurons by acting on the formation of the ionotropic glutamate receptors.

## RESULTS

### TDP-43^A315T^ expression does not significantly affect cortical neuron dendrite or axon outgrowth, but reduces dendritic arbor complexity

**Fig. 1.**
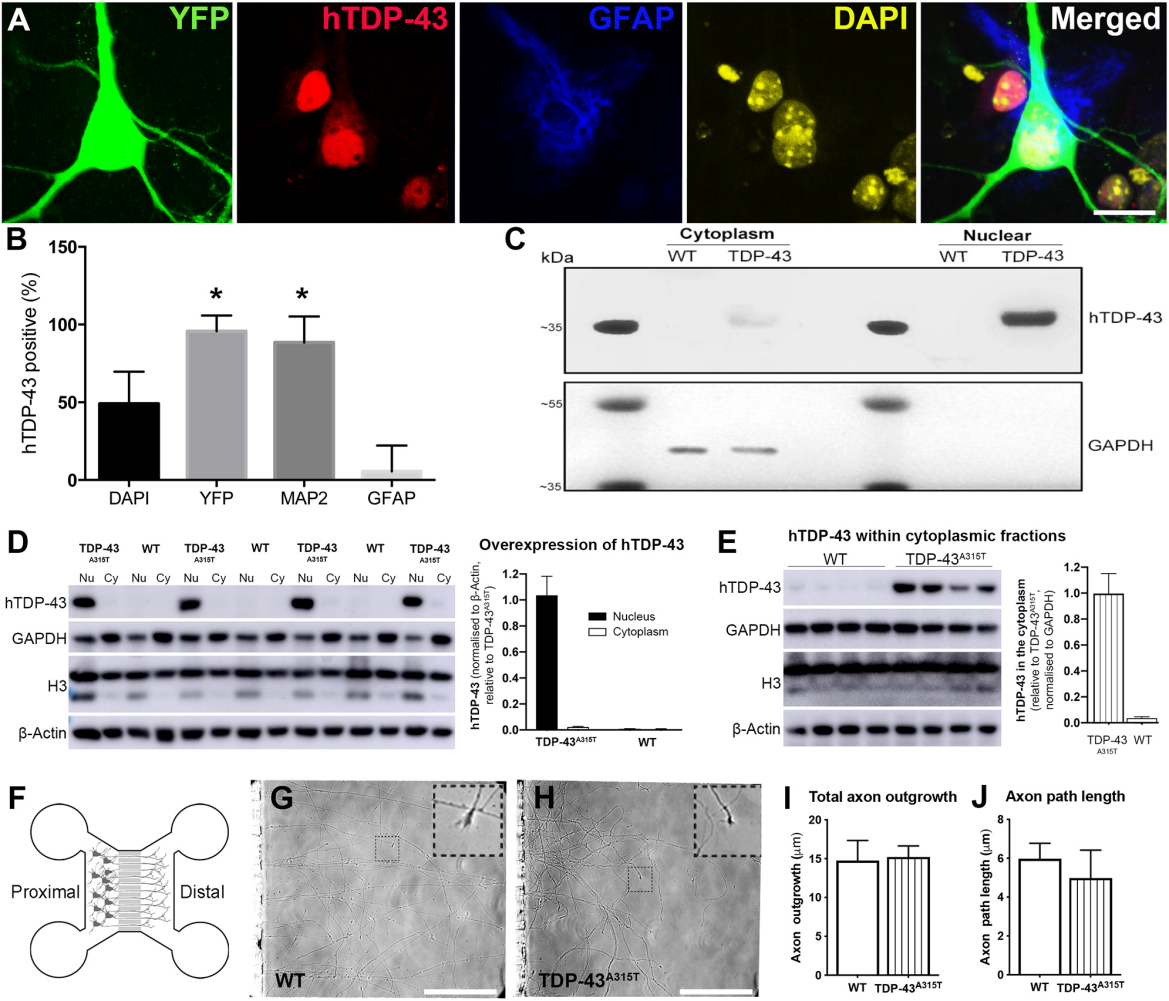
**Human TDP-43^A315T^ (hTDP-43^A315T^) expression in primary cortical neurons.** (A) Immunocytochemistry of YFP (green), hTDP-43 (red), GFAP (blue) and DAPI (yellow) in YFP:TDP-43^A315T^ cortical cultures at 10 DIV (*n=*3 biological replicates). (B) Quantification of the number of DAPI, YFP, MAP2 and GFAP cells positive for hTDP-43 demonstrated that ∼50% of DAPI positive cells were hTDP-43-positive, whereas ∼100% of YFP and MAP2 cells and very few GFAP cells were positive for hTDP-43. (C) Western blot of nuclear and cytoplasmic extractions of 15 DIV YFP:TDP-43^A315T^ cortical cultures. TDP-43^A315T^, detected with the antibody specific to hTDP-43, was predominately located in the nuclei of YFP:TDP-43^A315T^ cells; however, cytoplasmic localization was also observed. No hTDP-43 was detected in the cytoplasmic and nuclear compartments of YFP:WT cells, highlighting the specificity of the hTDP-43 antibody to the hTDP-43^A315T^ protein and confirming expression of hTDP-43^A315T^ in our cortical neuron culture model. (D) Quantification of hTDP-43 in 5 µg of cytoplasmic (Cy) and nuclear (Nu) protein fractions confirmed the specificity of hTDP-43 over-expression in TDP-43^A315T^ cortical neurons, and showed that cytoplasmic levels of hTDP-43 were ∼2% those of nuclear levels, with only negligible/background signal detected in WT neurons. (E) hTDP-43 was detected strongly in 20 µg of cytoplasmic protein from TDP-43^A315T^ neurons, with WT neurons showing negligible signal. (F) The microfluidic chamber culture platform isolates axons in the distal compartment from cell bodies in the proximal compartment. (G,H) Live microscopy images for axonal growth cones (inset) were captured at 6 DIV. (I,J) Tracking of total axon outgrowth and path length demonstrated no significant changes between YFP:WT and YFP:TDP-43^A315T^ neurons (*n*=99 axons and *n*=87 axons, respectively, altogether from three biological replicates). *P<0.05 (two-way ANOVA with Tukey's multiple comparisons test). Data are mean±s.e.m. Scale bars: 20 μm.

Synaptic loss is a major feature of neurodegenerative disorders, and to begin examining the effect of mutant TDP-43 on excitability we first examined the effect of TDP-43^A315T^ expression on the outgrowth of axons and dendrites, on which the pre- and postsynaptic compartments are located, respectively. YFP:TDP-43^A315T^ primary cortical neuron cultures were derived from single transgenic embryos. TDP-43^A315T^ expression was detected with an antibody specific to human TDP-43 (hTDP-43). To confirm which cells expressed hTDP-43, we immunostained and quantified cells double-labeled for neuronal and glial markers (Fig. 1). There were significantly more YFP-positive and MAP2-positive neurons with TDP-43^A315T^ expression than GFAP and DAPI positive cells [Fig. 1A,B; *F*(3,51)=68.84, *P*<0.0001, two-way ANOVA]. Very few GFAP-positive cells (astrocytes) were positive for hTDP-43 (Fig. 1B), confirming the neuronal expression of TDP-43^A315T^ in this model. To investigate the subcellular localization of mutant TDP-43^A315T^, cytoplasmic and nuclear extractions were performed at 15 days *in vitro* (DIV) (Fig. 1). TDP-43^A315T^ was predominately located in the nuclei of YFP:TDP-43^A315T^ cells (Fig. 1C,D); however, cytoplasmic localization was also observed (Fig. 1C,E). Negligible hTDP-43 was detected in the cytoplasmic and nuclear compartments of YFP:wild-type (WT) cells, which highlights the specificity of the hTDP-43 antibody to the hTDP-43^A315T^ protein and confirming expression of hTDP-43^A315T^ in the cortical neuron culture model (Fig. 1C-E). Disease models using TDP-43 knockout display significant alterations to the complexity of the lower motor neuron presynaptic terminal (Feiguin et al., 2009) and TDP-43 is localized and actively transported *in vivo* in motor neuron axons (Fallini et al., 2012). To investigate the effects of TDP-43^A315T^ on cortical axon outgrowth during neuronal development, cortical neurons from WT and TDP-43^A315T^ embryos were cultured in microfluidic chambers (Southam et al., 2013), which allows selective analysis of axons (Fig. 1F-H). There were no significant changes between YFP:WT and YFP:TDP-43^A315T^ neurons in total axon length or axon path length (Fig. 1I,J).

**Fig. 2.**
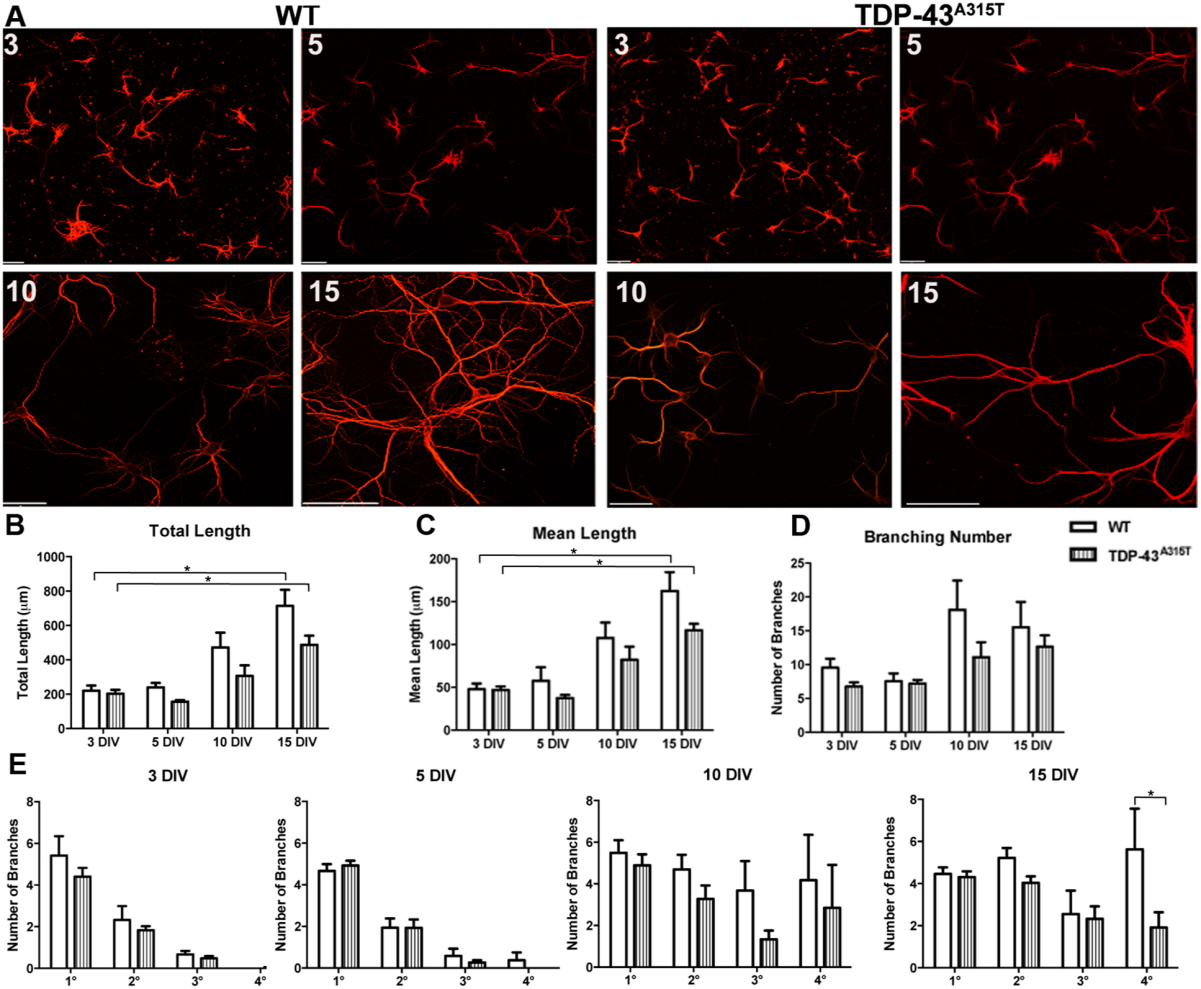
**The effect of TDP-43^A315T^ expression on dendrite development.** (A) Primary cortical neurons derived from YFP:WT (left) and YFP:TDP-43^A315T^ (right) single embryo cultures labeled for MAP2 at 3 DIV, 5 DIV, 10 DIV and 15 DIV. (B) Total dendrite length significantly increased between 3 and 15 DIV in both WT and TDP-43^A315T^ cultures. There was no significant difference in total dendrite length between WT and TDP-43^A315T^ at any time point. (C) Mean dendrite length significantly increased between 3 and 15 DIV in both WT and TDP-43^A315T^ cultures. There was no significant difference in mean dendrite length between WT and TDP-43^A315T^ at any time point. (D) There was no significant difference in dendritic branching number between 3 and 15 DIV in both WT and TDP-43^A315T^ cultures. There was no significant difference in dendritic branching number between WT and TDP-43^A315T^ neurons at any time point. (E) Quantification of the number of primary (1°) secondary (2°) tertiary (3°) and quaternary (4°) dendrite branches at 3, 5, 10 and 15 DIV demonstrated that TDP-43^A315T^ neurons became significantly less complex in quaternary branches by 15 DIV. *n=*3 biological replicates. *P<0.05 (two-way ANOVA with Tukey's multiple comparisons test). Data are mean±s.e.m. Scale bars: 20 μm.

Next, we investigated whether TDP-43^A315T^ affected dendrite development. To investigate dendrite outgrowth, using MAP2 immunoreactivity we measured the total dendrite length, mean dendrite length, dendritic branch number and dendritic branch order in YFP:WT and YFP:TDP-43^A315T^ neurons at 3, 5, 10 and 15 DIV (Fig. 2A). Total dendrite length significantly increased over time [*F*(3,43)=24.31, *P*<0.0001, two-way ANOVA], with greater total dendrite length at 15 DIV compared to 3 DIV in both YFP:WT and YFP:TDP-43^A315T^ neurons (YFP:WT, *P*<0.0001; YFP:TDP-43^A315T^, *P*<0.001; Tukey's multiple comparisons test) (Fig. 2A). There was no significant difference in total dendrite length between YFP:WT and YFP:TDP-43^A315T^ cortical neurons at any time point analyzed (Fig. 2B). Similarly, mean dendrite length significantly increased over time [*F*(3,43)=25.71, *P*<0.0001, two-way ANOVA], with greater mean length at 15 DIV compared to 3 DIV in both YFP:WT and YFP:TDP-43^A315T^ neurons (YFP:WT, *P*<0.0001; YFP:TDP-43^A315T^, *P*<0.001; Tukey's multiple comparisons test), but there was no significant difference in mean dendrite length between YFP:WT and YFP:TDP-43^A315T^ cortical neurons at any time point (Fig. 2C). To investigate dendrite complexity, the number of dendrite branches was quantified. There was no significant difference in dendritic branch number between YFP:WT and YFP:TDP-43^A315T^ neurons at any time point (Fig. 2D). Quantification of the number of primary (1°), secondary (2°), tertiary (3°) and quaternary (4°) dendrite branches at 3, 5, 10, and 15 DIV demonstrated that YFP:TDP-43^A315T^ neurons were less complex, with significantly fewer quaternary branches than YFP:WT neurons at 15 DIV (Fig. 2E) (*P*<0.05, Tukey's multiple comparisons test after two-way ANOVA).

### TDP-43^A315T^ significantly reduces dendritic spine density in cortical neurons

**Fig. 3.**
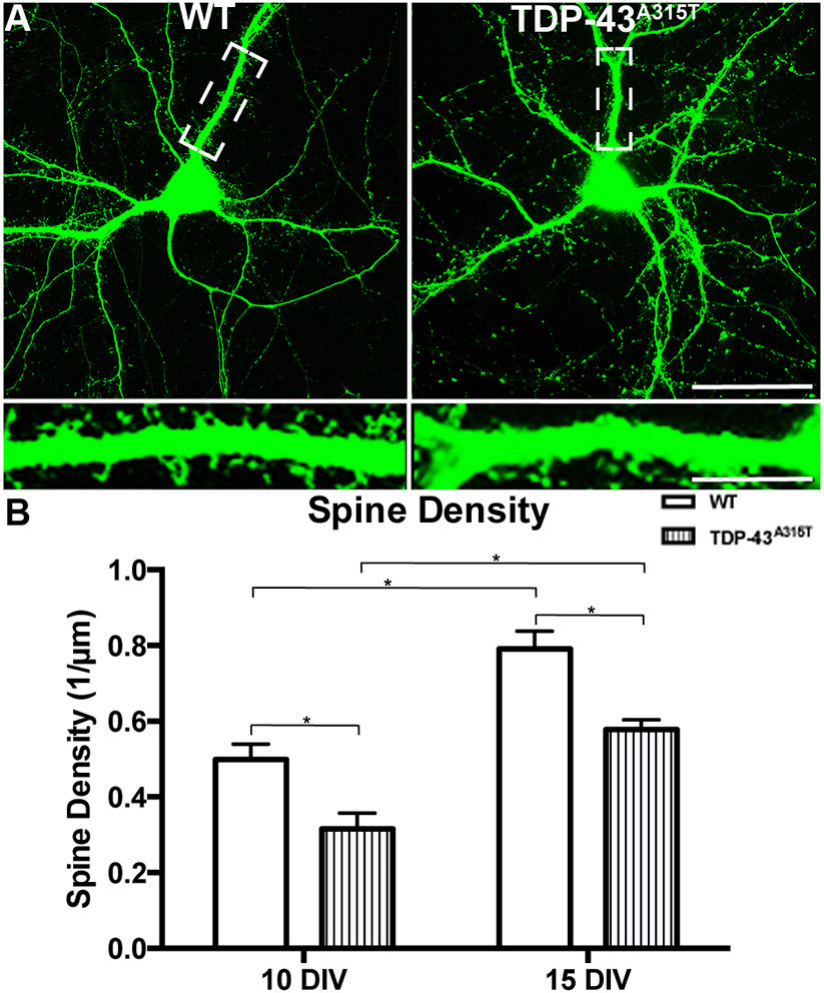
**The effect of TDP-43^A315T^ expression on dendrite spine development.** (A) YFP:WT and YFP:TDP-43^A315T^ cortical neurons at 15 DIV develop extensive dendritic spines (boxed areas, enlarged in insets). (B) Quantification demonstrated a significant increase in spine density between 10 and 15 DIV in both WT and TDP-43^A315T^ cortical neurons. There was a significant reduction in spine density in the TDP-43^A315T^ neurons at both 10 and 15 DIV in comparison to WT controls. *n=*3 biological replicates. *P<0.05 (two-way ANOVA with Tukey's multiple comparisons test). Data are mean±s.e.m. Scale bar: 50 μm; 20 μm in inset.

Dendritic spines are under constant turnover and morphological modification dependent on stimuli, environment and location; these capacities are key for neuronal connectivity and synaptic plasticity (Sala and Segal, 2014; Fiala et al., 2002). TDP-43 may have a role in the generation and maturation of dendritic spines, shown from *in vitro* studies using hippocampal neurons (Majumder et al., 2012; Wiens et al., 2005). Furthermore, we have shown that mutated TDP-43^A315T^ has a significant pathological effect on dendritic spine morphology and excitability in the motor cortex *in vivo* (Handley et al., 2017). To establish whether changes to dendritic spine density are present in an isolated cortical system *in vitro* similar to those found in the motor cortex *in vivo*, primary cortical neurons derived from YFP:TDP-43^A315T^ embryos were grown to 10 and 15 DIV. YFP:WT and YFP:TDP-43^A315T^ cortical neurons develop extensive dendritic spines by 10 and 15 DIV (Fig. 3A, inset). Spine density was increased by time in culture [*F*(1,45)=41.71, *P*<0.0001, two-way ANOVA] but decreased when the mutant TDP-43^A315T^ was expressed [*F*(1,45)=21.23, *P*<0.0001, two-way ANOVA], with a significant reduction in spine density in YFP:TDP-43^A315T^ neurons compared to YFP:WT controls at both 10 DIV and 15 DIV (10 DIV, *P*<0.05; 15 DIV, *P*<0.01; Tukey's multiple comparisons test) (Fig. 3B). This result indicates that loss of spine density is a dendrite-specific consequence of over-expression of human TDP-43 with the TDP-43^A315T^ mutation in our *in vitro* cortical neurons, and occurs independently of any gross changes in dendrite and axon development.

### TDP-43^A315T^ mutation alters of the expression of synaptic proteins in cortical neurons

**Fig. 4.**
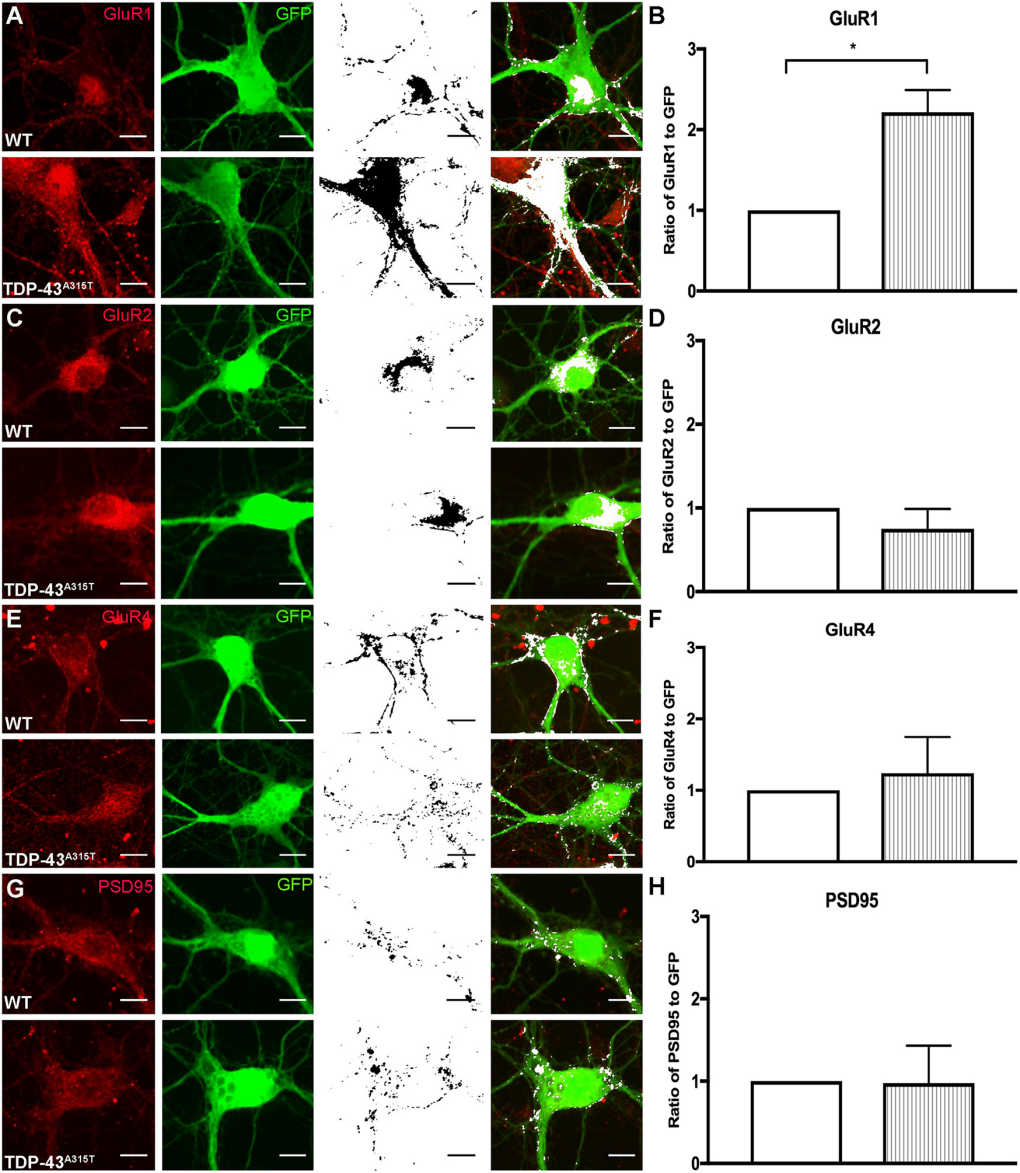
**The effect of TDP-43^A315T^ expression on synaptic receptor expression.** (A-H) YFP:WT and YFP:TDP-43^A315T^ primary cortical neurons at 10 DIV were labeled for postsynaptic receptors and GFP (antibody against GFP enhances endogenous YFP fluorescence). Immunocytochemistry labeling for the glutamate receptor subunits (red, first column) GluR1 (A), GluR2 (C) and GluR4 (E) and the postsynaptic protein PSD-95 antibody (G), and GFP antibody (green, second column); colocalization (black, third column) was identified in ImageJ and overlaid onto GFP fluorescence images (white; fourth column). (B,D,F) The ratio of GluR1 to GFP was significantly increased (B), whereas there was no significant differences in the ratio of GluR2 to GFP (D) and GluR4 to GFP (F) in the TDP-43^A315T^ neurons in comparison to WT controls. (H) There was no significant difference in the ratio of PSD-95 to GFP in the TDP-43^A315T^ neurons in comparison to WT controls. *n=*3 biological replicates. *P<0.05 (Student's *t*-test). Data are mean±s.e.m. Scale bar: 10 μm.

To determine whether TDP-43^A315T^-mediated spine loss is associated with alterations in the expression of postsynaptic proteins, colocalization between GFP (endogenous YFP fluorescence enhanced by anti-GFP antibody; denoted as GFP) and glutamate receptor subunits [GluR1 (also known as Gria1), GluR2 (Gria2) and GluR4 (Gria4)] or postsynaptic density marker PSD-95 was analyzed in YFP:WT and YFP:TDP-43^A315T^ cortical neurons grown to 10 DIV (Fig. 4). The glutamate receptor subunit GluR1 was significantly increased in the YFP:TDP-43^A315T^ neurons (Fig. 4A,B) [*t*(4)=4.450, *P*=0.0112, *t*-test]. There was no significant difference in GluR2, GluR4 and PSD-95 in the YFP:TDP-43^A315T^ neurons in comparison to the YFP:WT controls (Fig. 4C-H).

**Fig. 5.**
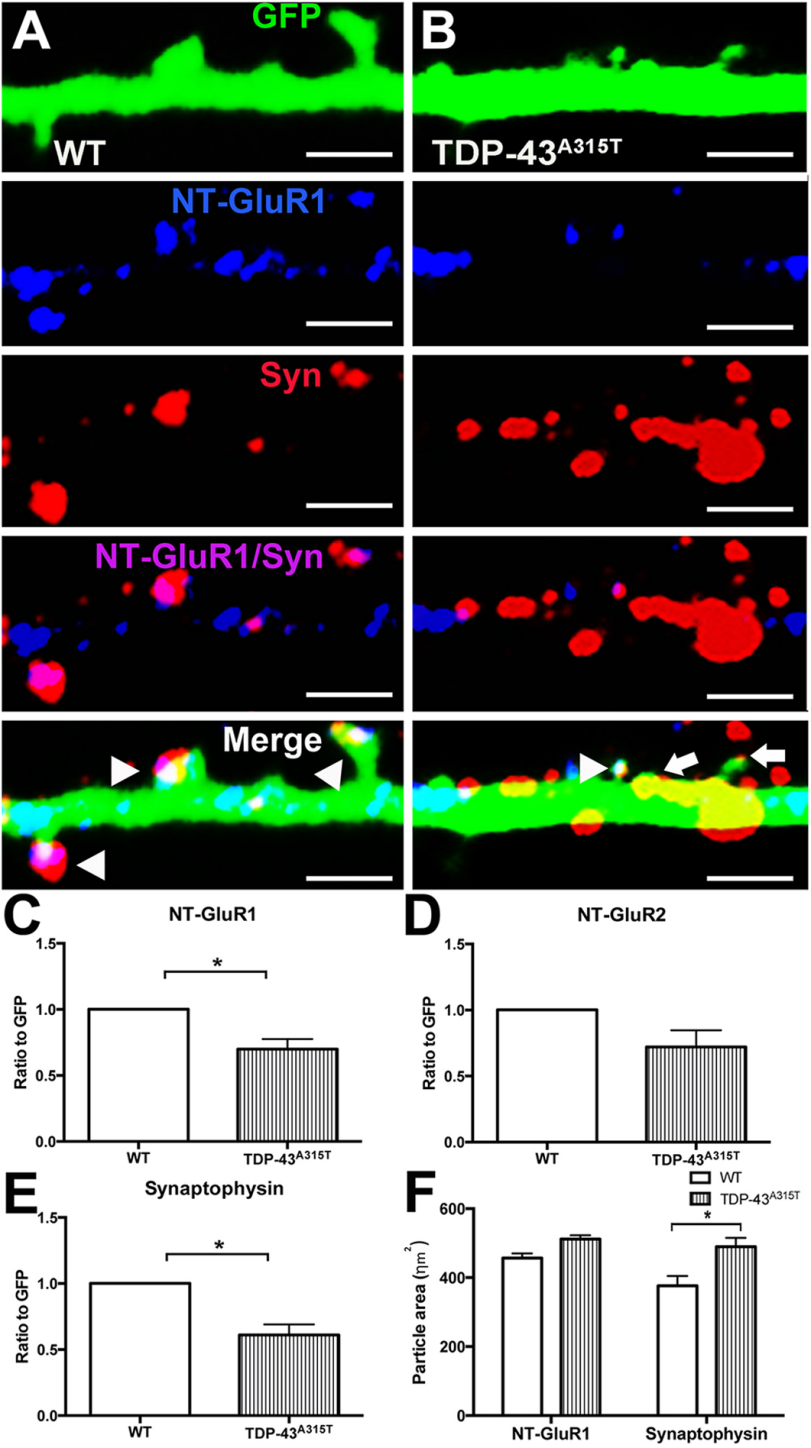
**The effect of TDP-43^A315T^ expression at the synapse.** (A) Immunocytochemistry for NT-GluR1 (blue), synaptophysin (Syn, red) and GFP (green) demonstrated that NT-GluR1 and synaptophysin colocalized at the dendritic spine (white) in YFP:WT dendrites (A, arrowheads). (B) In YFP:TDP-43^A315T^ dendrites, immunocytochemistry for NT-GluR1 (blue), synaptophysin (red) and GFP (green) demonstrated that spines were present that colocalized for NT-GluR1 and synaptophysin (white; arrowhead), with spines also present lacking NT-GluR1 (arrows); frequent accumulations of synaptophysin were observed outside the identified dendritic spines. (C) Quantification of the ratio of NT-GluR1 to GFP demonstrated significant decrease in the colocalization of NT-GluR1 in TDP-43^A315T^ cortical neurons in comparison to WT controls. (D) Quantification of the ratio of NT-GluR2 to GFP demonstrated no significant change in the ratio of colocalization of NT-GluR2 in TDP-43^A315T^ cortical neurons in comparison to WT controls. (E) Quantification of the ratio of synaptophysin to GFP showed significant decrease in the colocalization of synaptophysin in TDP-43^A315T^ cortical neurons in comparison to WT controls. (F) Quantification of the particle size of NT-GluR1 and synaptophysin demonstrated that there was a significant increase in the area of the synaptophysin particles in TDP-43^A315T^ cortical neurons in comparison to WT controls. *n=*3 biological replicates. *P<0.05 (Student's *t*-test and two-way ANOVA with Tukey's multiple comparisons test). Data are mean±s.e.m. Scale bars: 1 μm.

To investigate whether this change in the amount of GluR1 subunit affected its incorporation into AMPA receptors at the synapse, the presence of surface GluR1 in dendritic spines was investigated with an antibody against the extracellular N-terminal domain (NT-GluR1) in non-permeabilized neurons (Fig. 5). NT-GluR1 colocalized with the presynaptic marker synaptophysin in GFP-positive dendritic spines in YFP:WT neurons (Fig. 5A, arrowheads). However, in YFP:TDP-43^A315T^ neurons the presence of colocalized NT-GluR1 and synaptophysin within GFP-positive spines was reduced (Fig. 5B, arrows). GFP and NT-GluR1 colocalization at 10 DIV demonstrated there was a significant decrease of the ratio of NT-GluR1 to GFP in the TDP-43^A315T^ neurons (Fig. 5C) [*t*(4)=3.848, *P*=0.0183, *t*-test]. The ratio of NT-GluR2 to GFP in YFP:WT and YFP:TDP-43^A315T^ neurons was not different (Fig. 5D), indicating a selective decrease of GluR1 AMPA receptor subunits at the postsynapse. Quantification of the ratio of presynaptic protein synaptophysin to GFP demonstrated significant decrease in the overall colocalization of synaptophysin to GFP in YFP:TDP-43^A315T^ cortical neurons in comparison to YFP:WT controls [*t*(4)=4.809, *P*=0.0086, *t*-test] (Fig. 5E). Despite its lower overall presence, accumulations of synaptophysin were frequently observed in YFP:TDP-43^A315T^ dendrites, and quantification of the size of synaptophysin-immunoreactive particles confirmed a significant increase in the area of synaptophysin puncta in the YFP:TDP-43^A315T^ neuronal network in comparison to YFP:WT controls [*F*(1,74)=16.30, *P*=0.001, two-way ANOVA] with no change in NT-GluR1 particle area (Fig. 5F). This data indicates that despite increased overall levels of GluR1, TDP-43^A315T^ causes a decrease in the localization of GluR1 to the synapse and accumulation of synaptophysin at the presynaptic site, potentially driving a reduction in excitability in TDP-43^A315T^ cortical neurons.

### TDP-43^A315T^ mutation leads to hypoexcitability in cortical neurons

**Fig. 6.**
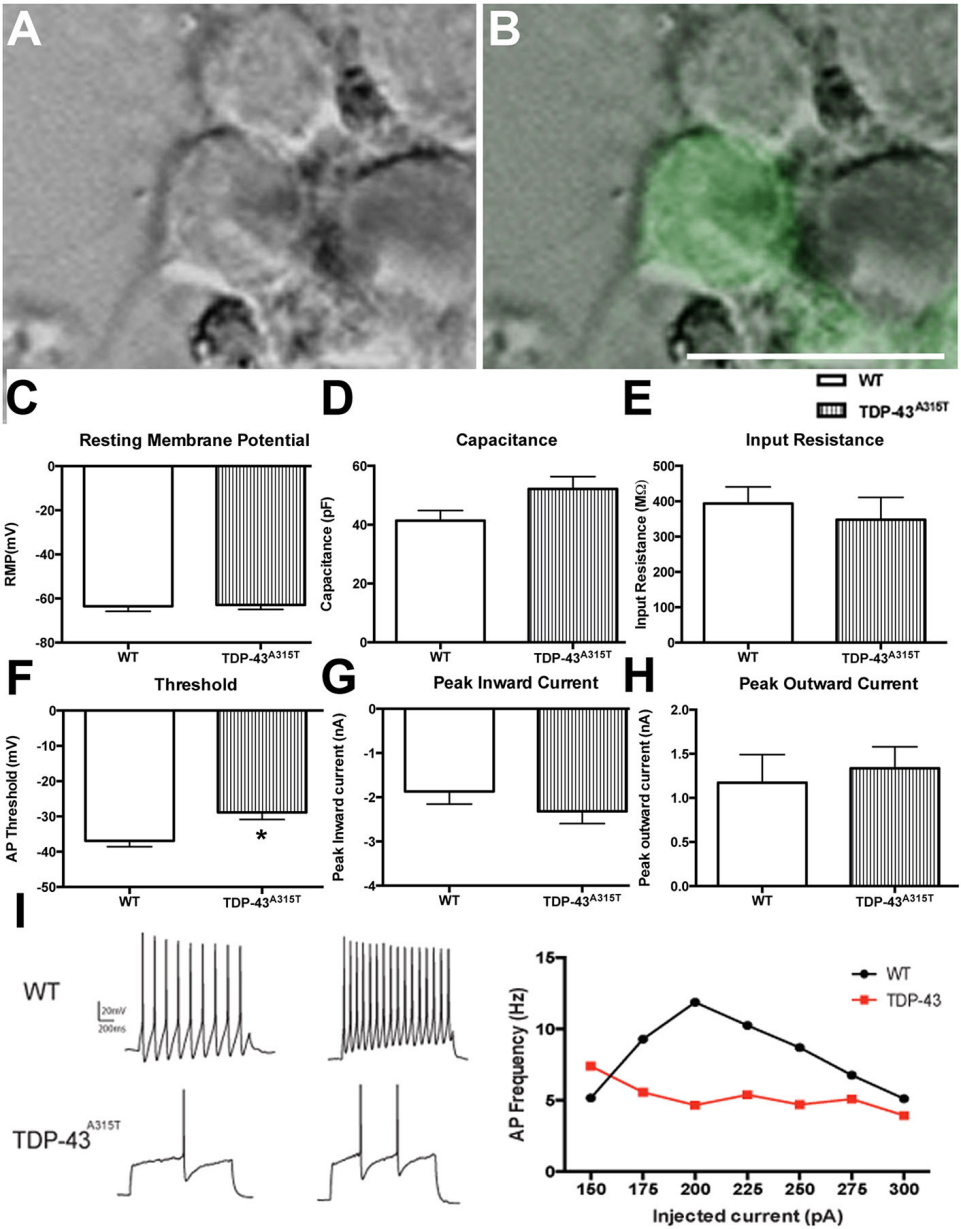
**The effect of TDP-43^A315T^ expression on neuronal excitability.** (A,B) Phase image of a TDP-43^A315T^ cortical neuron (A) that is YFP positive (B, green) selected for whole-cell patch clamp analysis at 10 DIV. Passive membrane parameters of YFP:WT and YFP:TDP-43^A315T^ cortical neurons were analyzed. (C-E) There were no significant differences in resting membrane potential (C), capacitance (D) and input resistance (E) between WT and TDP-43^A315T^ cortical neurons. (F) There was a significant increase in depolarization threshold in the TDP-43^A315T^ cortical neurons in comparison to WT controls. (G,H) There were no significant differences in peak inward (G) or peak outward (H) current. (I) Representative traces of WT and TDP-43^A315T^ patched neurons (left). Using a series of depolarizing current steps (100 to 500pA, in 25 pA increments, 200 ms duration), the (*f-I*) relationship generated indicated that the TDP-43^A315T^ neurons fired at lower rates for a given current compared to WT neurons (right). *n=*3 biological replicates. *P<0.05 (Student's *t*-test). Data are mean±s.e.m. Scale bar: 20 μm.

To determine whether the decrease in spine density reflects a decrease in synaptic transmission, we examined the electrophysiological properties of YFP:WT and YFP:TDP-43^A315T^ neurons *in vitro* at 10 DIV. YFP-positive neurons were used to make whole-cell patch clamp recordings (Fig. 6A,B). There were no significant differences in resting membrane potential (Fig. 6C), capacitance (Fig. 6D) and input resistance (Fig. 6E) between YFP:WT and YFP:TDP-43^A315T^ cortical neurons. However, there was a significant increase in depolarization threshold in the YFP:TDP-43^A315T^ cortical neurons in comparison to YFP:WT controls [*t*(18)=2.865, *P*=0.0103, *t*-test] (Fig. 6F). There were no significant differences in peak inward current (Fig. 6G) or peak outward current (Fig. 6H). The relationship between firing frequency and injected current (the *f-I* function) was also assessed to study the net excitability of cortical neurons with the TDP-43^A315T^ mutation. Using a series of depolarizing current steps (100 to 500 pA, in 25 pA increments, 200 ms duration), the *f-I* relationship that was generated indicated that the YFP:TDP-43^A315T^ neurons fired at lower rates for a given current compared to YFP:WT neurons (Fig. 6I). Despite similar passive membrane properties, YFP:TDP-43^A315T^ neurons fired significantly fewer action potentials during depolarization than YFP:WT neurons. Overall, these results indicate that the loss of GluR1 and concurrent loss of dendritic spines correlates to a decreased excitability of cortical pyramidal neurons *in vitro.*

## DISCUSSION

ALS is characterized by the progressive loss of motor neurons in the brain and spinal cord, with TDP-43 protein aggregation a consistent pathological hallmark. Changes in cortical excitability, and anterograde propagation of excitotoxicity through the motor system, are increasingly recognized as an important feature of ALS pathogenesis. Herein, we describe the alterations at the synapse of cortical pyramidal neurons caused by the A315T mutation in TDP-43. We demonstrate that dendritic spine density is significantly attenuated by the over-expression of human TDP-43^A315T^, preceding alteration in dendritic arbor complexity. Interestingly, despite upregulation of total GluR1, TDP-43^A315T^ causes a decrease in the localization of GluR1 to the synapse and accumulation of synaptophysin at the presynaptic site, with these synaptic alterations potentially driving the observed reduction in excitability in TDP-43^A315T^ cortical neurons. Together, these results provide a putative novel mechanistic link between mutation in TDP-43 and neuronal dysfunction in ALS.

Previous studies of pathogenic TDP-43 on dendritic and axonal structural integrity have yielded varying results. TDP-43 protein expression is autoregulated (Avendano-Vazquez et al., 2012; Ayala et al., 2011), and both over-expression and under-expression of TDP-43 can lead to selective neuronal vulnerability (Majumder et al., 2012). The manipulation of endogenous TDP-43 and WT hTDP-43 affects neurite outgrowth in different types of mammalian central nervous system neurons, and in the *Drosophila* peripheral nervous system (Yang et al., 2010; Fiesel et al., 2011; Iguchi et al., 2009; Liu-Yesucevitz et al., 2014; Fallini et al., 2012). In the present study, using a transgenic model of stable TDP-43^A315T^ expression, we found no significant changes in dendrite or axonal outgrowth. The absence of axonal outgrowth observed may be specific to cortical neurons, as previous studies in lower motor neurons have shown that mutant TDP-43 has a prominent effect on axons (Fallini et al., 2012). This suggests that the effect of TDP-43 over-expression and/or mutant TDP-43 may depend upon cell type. Interestingly, plasmid-mediated expression of mutant TDP-43^A315T^, TDP-43^Q331K^ or TDP-43^M337V^ in differentiated cortical neurons regulated overall neurite morphology relative to TDP-43^WT^ (Han et al., 2013). When investigating dendrite development through the branching order of dendrites, we found a loss of complexity by 15 DIV. Loss of dendrite arbor complexity can have dire effects on neuronal connectivity and has been shown to occur in neurodegenerative diseases such as Alzheimer's disease (Dorostkar et al., 2015). Our results demonstrate that, when identifying dendrites and axons separately, the TDP-43^A315T^ mutant specifically affects the structure of the dendritic arbor, potentially affecting the ability of TDP-43^A315T^ cortical neurons to receive and integrate incoming synaptic transmissions.

In hippocampal neurons, TDP-43 has been identified as a negative regulator of dendritic spine density (Majumder et al., 2012), acting together with Fragile X Syndrome protein FMRP to co-repress the translation of Rac1 mRNA (Majumder et al., 2016). Activation of the Rac1 pathway stabilizes dendritic spines and recruits AMPA receptors to the postsynaptic membrane, enhancing excitatory synaptic transmission (Wiens et al., 2005; Majumder et al., 2012). Consistent with a potential reduction in Rac1-mediated AMPA recruitment, in the present study we identified a loss of GluR1 at the postsynapse in TDP-43^A315T^ cortical neurons. The reduction of GluR1 at the postsynapse may result in attenuated receptivity of TDP-43^A315T^ cortical pyramidal neurons to glutamatergic inputs from surrounding neuronal cells, resulting in reduced excitability. However, we also identified an overall increase in GluR1 in the cytoplasm. Therefore, the changes observed indicate that either an overall increase in TDP-43 in the cytoplasm and synaptic compartments differentially affects the GulR1 regulation at the synapse, or that mutant TDP-43 may be producing a form of GluR1 that is less effective at being incorporated into the postsynapse membrane. The latter is plausible, as mutations in TDP-43 are known to affect its splicing function (White et al., 2018; Arnold et al., 2013). Furthermore, White et al. (2018) demonstrated with a TDP-43^Q331K^ knock-in mouse model that altered RNA splicing function may be due to a gain of TDP-43 function that has resulted from perturbed autoregulation.

Alterations in excitability are thought to play a key role in the pathogenesis of ALS. Clinical findings of cortical hyperexcitability (Eisen et al., 1993; Vucic et al., 2008; Bae et al., 2013), along with the prescribed ALS therapeutic agent Riluzole that is thought to act in part by dampening glutamatergic signaling (Doble, 1996), support the hypothesis of anterograde glutamate-mediated excitotoxicity as a primary cortical pathogenic origin for the disease (Eisen et al., 1993). Indeed, our previous research has shown the vulnerability of lower motor neurons to excitotoxicity (Blizzard et al., 2015; King et al., 2007). Pre-symptomatic cortical hyperexcitability can be detected not only in patients with ALS (Vucic et al., 2008; Menon et al., 2015), but also in pre-symptomatic SOD1^G93A^ and TDP-43^Q331K^ transgenic mouse models of disease (Kuo et al., 2004; Martin et al., 2013; Fogarty et al., 2015; Saba et al., 2015; Carunchio et al., 2010; Pieri et al., 2003; van Zundert et al., 2008; Fogarty et al., 2016). Synaptic GluR1 levels have previously been reported as increased in spinal cord motor neurons before disease onset in the human SOD1^G93A^ (hSOD1^G93A^) mouse (Zhao et al., 2008), initially suggesting that the increase could lead to subsequent glutamate-mediated excitotoxicity and ALS-like pathology. However, subsequent studies in the hSOD1^G93A^ mouse model showed that: disease-resistant motor neurons display early intrinsic hyperexcitability (Leroy et al., 2014); this hyperexcitability was absent from the adult spinal motor neurons (Delestrée et al., 2014) and was not present in vulnerable motor neuron populations at disease endstage (Fuchs et al., 2013); the inhibition of pre-symptomatic motor neuron excitability accelerated disease progression (Saxena et al., 2013). These later studies support a case for hypoexcitability to be the pathogenic mechanism in ALS. Our data may identify how AMPA receptor-mediated changes contribute to this. Specifically, in agreement with the earlier studies, we show an overall increase in GluR1 subunit, suggesting a potential increase in excitability. However, our surface receptor and functional studies have also identified that, although GluR1 expression may be increased, its vital localization to the postsynaptic membrane is decreased, with an overall decrease in excitability. Therefore, we have identified that the pathogenic cascade that is initiated by hypoexcitability in ALS may be occurring at the postsynaptic AMPA receptor.

The advent of iPSC studies has provided some clarity over whether patient-derived neurons display an intrinsic excitability alteration. At early time points in culture, motor neurons derived from *SOD1*-ALS, *C9ORF72*-ALS and *FUS*-ALS patients showed hyperexcitability (Wainger et al., 2014), whereas a separate study showed motor neurons derived from *C9ORF72*-ALS patients exhibited hypoexcitability at a later time point in culture (Sareen et al., 2013). Subsequently, it was shown that motor neurons derived from *TARDBP*-ALS and *C9ORF72*-ALS patients demonstrated an initial phase of hyperexcitability, followed by a progressive loss of both action potential output and synaptic activity (Devlin et al., 2015). This shift from a hyperexcitable phenotype to a hypoexcitable phenotype, although potentially representing both initiating factors and compensatory mechanisms, indicates that the transition towards hypoexcitability may be a key feature in the pathogenic decline of motor neurons. The data from our current study demonstrates hypoexcitability mediated by TDP-43^A315T^ in 10 DIV cortical pyramidal neurons. Although it is difficult to determine the stage of disease in ALS patients to which cultured primary neurons correspond, and investigation of earlier or later time-points is technically fraught because of the nature of primary rodent neurons *in vitro*, the hypoexcitable phenotype we report here may correspond to that noted in iPSC-derived neurons maintained in culture over an extended period of time (Devlin et al., 2015; Sareen et al., 2013). Furthermore, although we found changes in depolarization threshold related to the mutation, there were no alterations in input resistance. Changes in action potential threshold are often, but not always, related to changes in the input resistance of a neuron. This is because spike threshold as measured at the soma is also dependent on the active properties of the membrane, and because it happens at the axonal initial segment, many other variables can have an effect, such as the density and spatial distribution of voltage-gated sodium and potassium channels in the initial axon segments and the presence and extent of dendritic action potentials (Goldwyn and Shea-Brown, 2011; Platkiewicz and Brette, 2010; Platkiewicz and Brette, 2011; Yi et al., 2015). The specific involvement of any of these factors in the electrophysiological changes found in the TDP-43 neurons would need to be further investigated.

In the present study, we have identified that TDP-43^A315T^ cortical projection neuron hypoexcitability was associated with loss of the AMPA receptor subunit GluR1 at the dendritic spine. Interestingly, enhancing excitability within a vulnerable motor neuron population in hSOD1^G93A^ mice was neuroprotective to the point of reversing disease pathology (Saxena et al., 2013). As such, a carefully targeted therapy designed to increase the incorporation of GluR1 subunits into the postsynaptic membrane in cortical projection neurons may be successful in restoring appropriate cortical neuron excitability in these particular cells. However, further research into temporal and cell-type-specific changes in excitability in ALS cortical circuits, and the presence of compensatory mechanisms in the surrounding cortical network, is required in order to determine the potential success of such a therapy.

In summary, in the current study we have identified that TDP-43^A315T^ reduces GluR1 at the postsynapse and attenuates the intrinsic excitability of cortical pyramidal neurons, providing a link between the pathological effect of TDP-43 mutation and attenuated neuronal synaptic function. Identification of the timing of neuronal hypoexcitability within ALS patient disease progression, the exact mechanisms by which mutant TDP-43 lowers synaptic GluR1, and modulation of synaptic GluR1 levels, may provide novel therapeutic targets for the treatment of this devastating neurodegenerative disease.

## MATERIALS AND METHODS

### Ethical statement

All experiments performed on animals were approved by the University of Tasmania Animal Ethics Committee (A0014118) and conducted according to the Australian Code of Practice for the care and use of animals for scientific purposes. Animals were bred at the University of Tasmania's animal facility and housed in individually ventilated cages in a temperature-controlled environment, with a 12-h light/dark cycle and access to food and water *ad libitum*.

### Transgenic mice

*Thy1*-YFP-16 transgenic mice (JAX 003709; The Jackson Laboratory) express cytosolic YFP under the control of the pyramidal/projection-neuron-specific *Thy-1* promoter (Feng et al., 2000). *Prp*-TDP-43^A315T^ transgenic mice (JAX 010700; The Jackson Laboratory) express hTDP-43 with a 315 A-T substitution mutation (TDP-43^A315T^) (Wegorzewska et al., 2009; Herdewyn et al., 2014). These two transgenic mouse lines were time-mated to generate YFP:TDP-43^A315T^ transgenic embryos and YFP:WT controls.

### Primary neuronal culture

Primary dissociated cortical neuron cultures from mouse embryos were prepared as previously described (Blizzard et al., 2013; Brizuela et al., 2017; Brizuela et al., 2015). Briefly, *Thy1*-YFP-16 female mice that had been time-mated with male *Prp*-TDP-43^A315T^ mice were euthanized by CO_2_ exposure at 15.5 days of gestation. Embryos were genotyped for YFP-transgenic status by fluorescent imaging using 470 nm light on a Carestream Image Station 4000MM Pro (Carestream Molecular Imaging). YFP-positive embryos were used to generate three biological replicates for cortical neuron culture, and tails from the respective embryos were kept for TDP-43^A315T^ genotyping. The top layer of the cortex was removed from single embryos and dissociated by 5 min enzymatic digestion at 37°C (0.025% w/v trypsin in 5 ml Hank's Buffered Salt Solution; both Thermo Fisher Scientific), which was halted by the addition of 1 ml of pre-warmed initial medium (Neurobasal supplemented with 2% v/v B27, 10% v/v fetal calf serum, 0.5 mM L-glutamine, 25 μM glutamic acid and 1% v/v antibiotic-antimycotic; Thermo Fisher Scientific). Cells were triturated and cell viability and concentration were assessed using Trypan Blue vital dye exclusion. Cells were plated into 24-well plates, which contained coverslips that had been pre-coated with 0.001% poly-L-lysine (Sigma-Aldrich) in 0.01 M PBS, or into 6-well plates and kept in a humidified incubator at 37°C and 5% CO_2_. At 1 DIV, initial medium was replaced with serum-free growth medium (Neurobasal supplemented with 2% v/v B27, 0.5 mM L-glutamine, 1% v/v antibiotic-antimycotic); after that, ∼50% of the medium was replaced every 2-3 days. All reagents were authenticated and tested for contamination. No randomization was performed; however, it was ensured that multiple single cell cultures were used from a minimum of three different litters.

### TDP-43^A315T^ genotyping

REDExtract-N-Amp Tissue PCR Kit (Sigma-Aldrich) was used to isolate total DNA from tails. Primer sequences were: hTDP-43 forward GGATGAGCTGCGGGAGTTCT, reverse TGCCCATCATACCCCAACTG; and T-cell receptor alpha/delta (DNA control gene) forward CAAATGTTGCTTGTCTGGTG, reverse GTCAGTCGAGTGCACAGTTT. PCR products were separated on a 1.5% agarose gel and visualized under UV light (Carestream Image Station).

### Immunocytochemistry and morphological analyses

Cultures at 3 DIV, 5 DIV, 10 DIV and 15 DIV were fixed in 4% w/v paraformaldehyde in PBS before immunolabeling. Primary antibodies (see Table S1) were diluted in 0.3% v/v Triton X-100 (Sigma-Aldrich) in PBS (except for NT-GluR1 and NT-GluR2, which were diluted in PBS without Triton X-100), and applied for 1 h at room temperature followed by overnight incubation at 4°C. Coverslips were washed thrice in PBS before the application of isotype- and species-specific secondary antibodies (see Table S1), diluted 1:1000 in 0.01 M PBS and applied to coverslips for 1.5 h at room temperature. Coverslips were washed thrice with PBS before mounting in fluorescent mounting medium (Thermo Fisher Scientific). Immunolabeling was visualized and imaged using an UltraView spinning-disc confocal microscope with Velocity Software (Perkin Elmer).

For dendrite structure analysis, cells in confocal immunolabeled images were traced using Neurolucida software (MBF Bioscience). Branched structure analysis was used to analyze the length, the branching number and the number of primary, secondary, tertiary and quaternary order dendrites of MAP2-labeled neurons. Dendrites were traced and spines were marked on YFP-positive neurons, with tracings exported to Neurolucida Explorer 11 (MBF Bioscience) for spine quantification. The spine analysis was performed by an investigator blinded to genotype.

### Quantitation of colocalization

Primary cortical neurons fixed at 10 DIV were used to investigate expression and localization of synaptic proteins. Postsynaptic receptors and synaptophysin (for antibodies see Table S1) were immunolabeled then imaged using an UltraView spinning-disc confocal microscope. The ratio of receptor expression to YFP was analyzed using the ImageJ colocalization plugin (Schindelin et al., 2012), and the ratio for WT neurons was normalized to 1 for analysis.

### Electrophysiology

At 10 DIV, cortical neurons that had been cultured on glass coverslips were superfused at 24±1°C with bicarbonate-buffered solution (155 mM NaCl, 3.5 mM KCl, 2 mM CaCl_2_, 1.5 mM MgCl_2_, 10 mM glucose; osmolarity adjusted to 305-310 mOsm/kg by the addition of d-sorbitol; all Sigma-Aldrich). Patch electrodes formed from borosilicate glass capillaries (1.5 mm outer diameter ×0.86 mm inner diameter) (Harvard Apparatus) with a resistance of 7-9 MΩ were filled with a solution containing 130 mM K-gluconate, 4 mM NaCl, 10 mM HEPES, 0.5 mM CaCl_2_, 10 mM BAPTA, 4 mM MgATP, 0.5 mM Na_2_GTP 0.5 (pH 7.4, set with KOH) and osmolarity adjusted to 290 mOsm/kg with d-sorbitol. An Axopatch 200B amplifier (Molecular Devices) was used for voltage- and current-clamp. Data was adjusted to account for the electrode-junction potential. Data were sampled at 10 kHz and filtered at 5 Hz using pClamp 9.2 software (Molecular Devices), and offline analysis was performed using Igor (WaveMetrics) and Axograph.

### Nuclear and cytoplasmic protein extraction

Nuclear and cytoplasmic protein was extracted using a NE-PER Nuclear and Cytoplasmic Extraction Reagents kit (Thermo Fisher Scientific). Briefly, cortical cultures (6×10^5^ cells) were grown on 6-well plate to 15 DIV. At 15 DIV, cells were removed from the plate using a cell scraper, in PBS, and then centrifuged at 500 ***g*** for 5 min. Cells were resuspended in PBS and transferred (2×10^6^) to a 1.5 ml microcentrifuge tube and repelleted at 500 ***g*** for 2-3 min. Cytoplasmic and nuclear extract was isolated as per the protocol.

### Western blotting

Protein concentration of nuclear and cytoplasmic protein fractions was determined using the DC Protein Assay (Bio-Rad). Nuclear and/or cytoplasmic fractions were solubilized in Laemmli buffer (Bio-Rad) with 5% β-mercaptoethanol (Bio-Rad) and separated by size using NuPAGE 4-12% Bis-Tris gels with MES SDS running buffer, alongside PageRuler Plus pre-stained protein ladder (all Life Technologies). Proteins were transferred to PVDF membrane using NuPAGE transfer buffer (Life Technologies) with 10% ethanol (Sigma-Aldrich). Membranes were blocked using 5% w/v non-fat milk powder in Tris-buffered saline with 0.05% Tween-20 (Sigma-Aldrich) (TBS-T), then incubated with primary antibodies overnight at 4°C (Table S1), washed in TBS-T, incubated for 1 h with secondary antibody at room temperature (Table S1) and washed in TBS-T. Membranes were then imaged using Amersham Imager 600 (GE Lifesciences) with Immobilon chemiluminescence substrate (Millipore). Membranes were sequentially re-probed after incubation in Restore PLUS Western Blot Stripping Buffer (Thermo Fisher Scientific). Quantification of western blot bands was performed with integrated density values obtained using ImageJ.

### Axonal outgrowth analysis

Cortical neurons (5×10^5^ cells) were plated into the somal compartment of microfluidic chambers (Southam et al., 2013) on square coverslips pre-coated with 0.001% poly-L-lysine. At 6 DIV, time-lapse images of the microfluidic chamber axonal compartment were obtained (one image every 2 min for 30 min) using a Nikon TiE motorized inverted microscope with ×40/0.95 objective (Nikon Instruments). Axon outgrowth and path length were calculated using NIS-Elements AR 3.2 software (Nikon Instruments).

### Statistical analysis

All statistical analysis was performed in GraphPad Prism (GraphPad Software) using *t*-tests and one-way or two-way ANOVA with Tukey's post-hoc correction for multiple comparisons. *P*<0.05 was considered significant. Data is reported as mean±s.e.m. Where appropriate, the person performing the neurite and dendrite spine tracing was blinded to genotype.
